# High-throughput and site-specific identification of 2′-*O*-methylation sites using ribose oxidation sequencing (RibOxi-seq)

**DOI:** 10.1261/rna.061549.117

**Published:** 2017-08

**Authors:** Yinzhou Zhu, Stephan P. Pirnie, Gordon G. Carmichael

**Affiliations:** Department of Genetics and Genome Sciences, UConn Health, Farmington, Connecticut 06030, USA

**Keywords:** 2′-*O*-methylation, RNA editing, RNA modification, ribosomal RNA

## Abstract

Ribose methylation (2′-*O*-methylation, 2′-*O*Me) occurs at high frequencies in rRNAs and other small RNAs and is carried out using a shared mechanism across eukaryotes and archaea. As RNA modifications are important for ribosome maturation, and alterations in these modifications are associated with cellular defects and diseases, it is important to characterize the landscape of 2′-*O*-methylation. Here we report the development of a highly sensitive and accurate method for ribose methylation detection using next-generation sequencing. A key feature of this method is the generation of RNA fragments with random 3′-ends, followed by periodate oxidation of all molecules terminating in 2′,3′-OH groups. This allows only RNAs harboring 2′-OMe groups at their 3′-ends to be sequenced. Although currently requiring microgram amounts of starting material, this method is robust for the analysis of rRNAs even at low sequencing depth.

## INTRODUCTION

A great majority of 2′-*O*-methylations are directed by Box C/D snoRNAs, noncoding RNAs that guide the modification of target sites via complementary RNA sequences. In humans, snoRNAs are assembled into snoRNP particles, containing the conserved core proteins NOP56, NOP58, fibrillarin (the catalytic component), and 15.5K (Tycowski et al. 1996; Filipowicz and Pogači 2002; Watkins and Bohnsack 2012). 2′-*O*-methylation has been extensively studied for a number of years with the goal of establishing functional and mechanistic links between this modification with specific biological pathways. Early studies demonstrated that 2′-*O*-methylations on rRNAs are indispensable for ribosome biogenesis (Tollervey et al. 1993); 2′-*O*-methylation has also been shown to be present on tRNAs and has been implicated to be crucial in translational circuitries (Satoh et al. 2000; Guy et al. 2015). A substantial portion of known methylated sites in rRNA lie in close proximity to ribosome functional sites such as regions around the peptidyl transfer center, suggesting the potential involvement of such modifications in rRNA folding, stability, and translation (Decatur and Fournier 2002). Ribose methylated bases are also found at mRNA caps and are involved in host pathogen responses (Daffis et al. 2010; Rimbach et al. 2015). Recent evidence indicates that in addition to being associated with the 5′ cap, mRNAs might potentially possess internal 2′-*O*-methylated sites (Lee et al. 2016).

The list of known 2′-*O*-methylation sites is frequently updated, as experimental techniques evolve and mature. However, until recently, a major hurdle in obtaining a more complete profile of the 2′-*O*-methylation landscape has been the lack of an efficient and reliable modification-specific and high-throughput detection method. Methylation sites have traditionally been mapped using targeted approaches including primer extension under limiting dNTP concentrations, where reverse transcriptase stalls when encountering a methylation site, or resistance to RNaseH digestion when synthesized DNA oligos are introduced (Yu et al. 1997; Maden 2001). Primer extension experiments are particularly prone to false positives for detecting 2′-*O*-methyl sites due to nonspecific polymerase pausing or secondary structure-induced pausing, and more laborious mass spectrometry is required to confirm the detection (Qiu and McCloskey 1999). Primer extension is also not suitable for de novo site detection and high-throughput screening, because the base position needs to be known for primer design. This makes primer extension most useful only as a confirmation tool. This method has, however, as described in RIM-seq and 2OMe-seq, recently been adapted for high-throughput detection of ribose methylation sites by combining random priming with next-generation sequencing (Incarnato et al. 2016; Jorjani et al. 2016). This study identified over 400 sites, almost 300 more than what have been curated in human rRNAs (Lestrade and Weber 2006). It is unclear, however, how many of the novel sites are true positives, owing to an inherent high false-positive rate of primer extension. Although potential matches to BoxC/D snoRNAs were bioinformatically identified for some of the novel sites found in the study, methylation was not confirmed, because there are known snoRNAs that interact with targets without guiding the deposition of methyl on ribose (Cavaillé and Bachellerie 1998; Lafontaine 2015). Furthermore, non-RNA-guided 2′-*O*-methylation has been reported in mice and it is possible that other mammals may share this feature (Kirino and Mourelatos 2007). As a consequence, these sites might not accurately represent the methylation pattern until they are further validated.

Ribometh-seq, another high-throughput method aimed at detecting 2′-*O*-methylation sites, has surfaced recently and utilizes the property of resistance to alkaline hydrolysis of ribose methylated bases. Thus, by randomly hydrolyzing RNA and performing next-generation sequencing at very high depth, there should be uniform coverage of 3′-end positions across regions of interest except at positions of 2′-*O*-methylation. This method overall has much better specificity and accuracy, as it has successfully detected about the same number of sites in rRNAs as have been annotated. Several novel sites were also validated by mass spectrometry (Krogh et al. 2016). However, the method relies heavily on negative rather than positive signals. In addition, the requirement for high read depth and coverage makes such studies costly, and the method can also suffer from high background noise due to resistance to alkaline hydrolysis of highly structured regions (Marchand et al. 2017). In order to address these issues, we have developed a 2′-*O*-methyl ribose-specific, high-throughput method, which relies on positive rather than negative signals, to detect 2′-*O*-methylation sites.

## RESULTS AND DISCUSSION

### Key principles of RibOxi-seq

**FIGURE 1.**
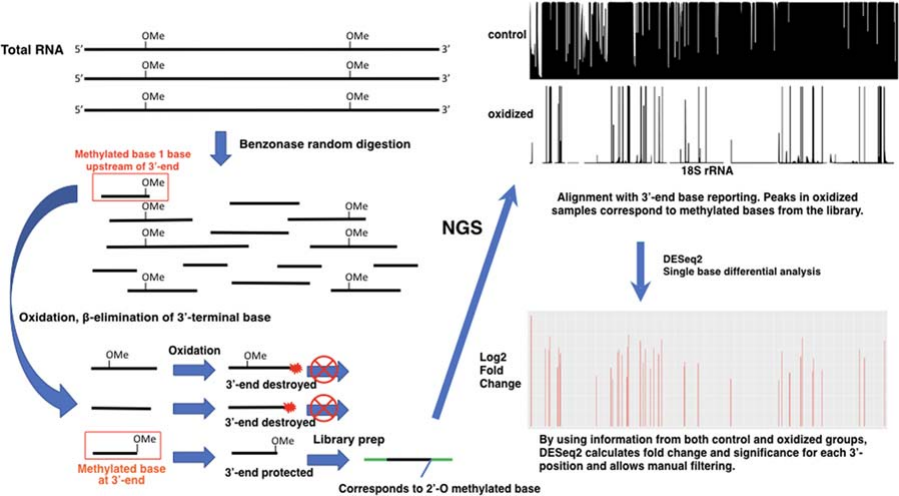
Work flow of the RibOxi-seq method. As shown on the *left*, rRNAs are digested randomly with Benzonase to generate RNA fragments that have 2′,3′-OH ends. Because the fragments are relatively small, some of those containing 2′-OMe will have methylated bases near their 3′-ends. β-Elimination is performed on the RNA fragments from the previous step to expose ribose methylated bases to the very 3′-ends. The resulting RNA pool is then oxidized so that fragments with methylated bases at the 3′-ends are protected. The fragments with ribose methylated bases at the 3′-end are available for linker ligation and are therefore enriched for RNA-seq library construction. After sequencing, data are processed and mapped to visualize the alignment of 3′-end bases to a reference genome in the UCSC Genome Browser (*top right*). The data are statistically analyzed using DESeq2 for enrichment, and significance for each base position is shown in the *bottom right*. (Boxed in red) Fragments susceptible to sequencing.

**FIGURE 2.**
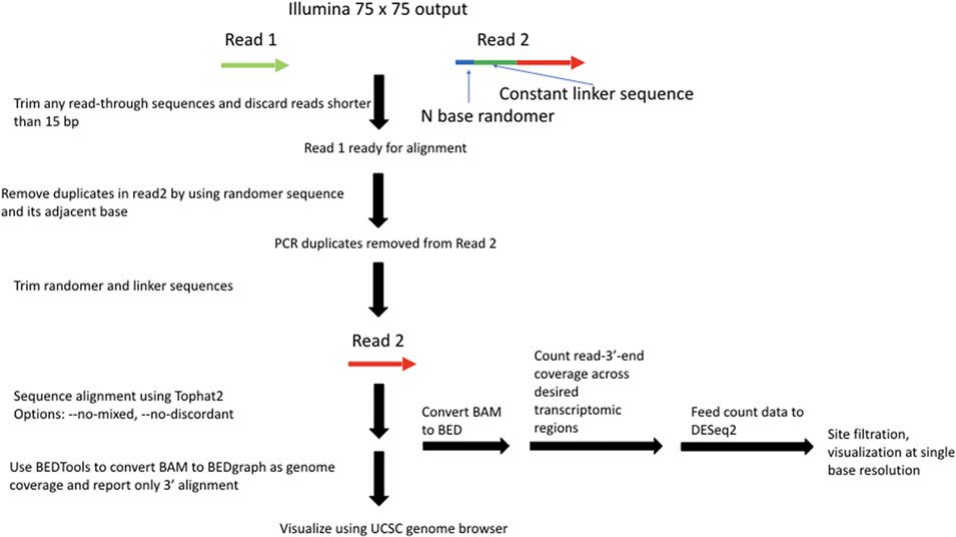
RibOxi-seq data processing and analysis pipeline.

**FIGURE 3.**
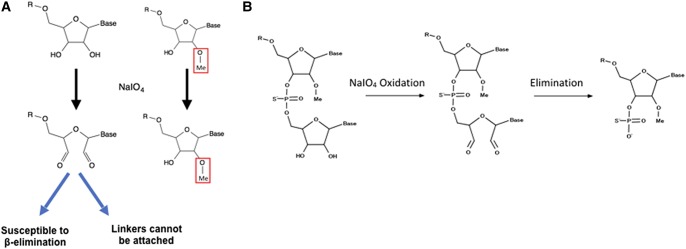
(*A*) Oxidation of RNA 3′-ends by sodium periodate (NaIO_4_). RNA fragments that are terminally methylated are protected from oxidation, while nonmethylated fragments will have their ends oxidized into dialdehyde, thus losing reactivity to ligation reactions. (*B*) To expose 2′-*O*-methylated bases at the 3′-terminus, at least one round of β-elimination is required. RNA fragments are first oxidized, then β-elimination catalyzes the leaving of 3′-terminal bases.

The key feature of the RibOxi-seq method is the preparation of fragmented RNAs containing 3′-ends that are either unmethylated or 2′-*O*-methylated. Then, an oxidation step renders the nonmethylated ends incapable of ligation to linkers used for high-throughput library construction. After sequencing, the reads are aligned to a reference genome and only positions of the 3′-ends of aligned fragments are counted and displayed for each base position. The count data for oxidized and nonoxidized samples are then normalized, compared, and analyzed using DESeq2 for single-base resolution methylation site determination (Figs. 1, 2). The major difference between our method and currently available methods is its specificity and its reliance on positive rather than negative signals. In the RibOxi-seq method, the 2′ and 3′ hydroxyls of non-2′-*O*-methylated riboses are converted into dialdehydes using sodium periodate (NaIO_4_), thus preventing them from being ligated to linkers for sequencing library construction (Fig. 3A). Before this critical step, however, it is important to ensure that all possible 3′-ends are represented in the samples to be analyzed. While in theory this can be achieved simply by randomly digesting total RNA to generate small fragments, currently available methods do not readily expose 2′-*O*-methylated 3′-ends because 2′-*O*-methylated bases are resistant to nuclease or alkaline cleavage (Maden et al. 1995; Maden 2001). In the RibOxi-seq method, we use Benzonase nuclease for the first step of RNA degradation. The advantage of this step over alkaline hydrolysis is that Benzonase leaves 3′-ends that lack phosphates, thus eliminating an extra dephosphorylation step. However, like other nucleases, Benzonase is not able to cleave 5′–3′ phosphodiester bonds of 2′-*O*-methylated bases (Supplemental Fig. S1). As a consequence, the nearest possible position for a 2′-*O*-methylated base is one base upstream of the original 3′-end of an RNA fragment (Fig. 1). Thus, an additional step is required to remove at least one base from 3′-ends to expose methylated bases before final oxidation can be effective (Fig. 3B). Alefelder et al. (1998) used β-elimination to chemically remove oxidized RNA terminal oligonucleotides for the purpose of studying RNA oligonucleotide structure. We adapted their chemical reactions such that we first fragment RNAs to produce ends containing 2′,3′-OH groups. These are oxidized using NaIO_4_, and β-elimination is then carried out under alkaline conditions to remove the terminal oxidized RNA nucleotide, resulting in mixtures of RNA with either 2′-*O*-methylated or nonmethylated bases at their 3′-ends (Fig. 3B). After this step, terminal 2′-*O*-methylated bases possess 3′-phosphates, while nonmethylated bases have a mixture of cyclic-phosphates, 3′-phosphates, and 2′-phosphates. Like 2′-*O*-methylated bases, the 2′-phosphate and cyclic-phosphates on nonmethylated bases render these ends resistant to subsequent oxidation. Most available phosphatases, such as calf intestine alkaline phosphatase, are only active in removing 3′-phosphates. If used, nonmethylated bases with 2′-phosphates will survive the oxidation step and generate false-positive signals in sequencing. To prevent this phenomenon, T4 polynucleotide kinase (T4 PNK) is used. Although less efficient than other widely used phosphatases, the advantage is that this enzyme nevertheless is capable of removing all three types of phosphates under acidic conditions in the absence of ATP (Das and Shuman 2013). It has been shown that T4 PNK's phosphatase activity is sufficient to remove the majority of the phosphates in <40 min at 37**°**C (Honda et al. 2016). Since RNA fragments with phosphates can generate bias in subsequent steps, we have increased enzyme concentration and extended incubation time to 4 h. With proper end treatment, the mixtures generated after β-elimination and T4 PNK treatment contain fragments with ends of either 2′,3′-OH or 2′-OMe,3′-OH. The subsequent round of NaIO_4_ oxidation thus enriches 2′-*O*-methylated ends as only these fragments remain intact and are available for subsequent 3′-linker ligation and sequencing library construction.

### RibOxi-seq accurately identifies annotated 2′-*O*-methylation sites within 18S and 28S rRNAs

**FIGURE 4.**
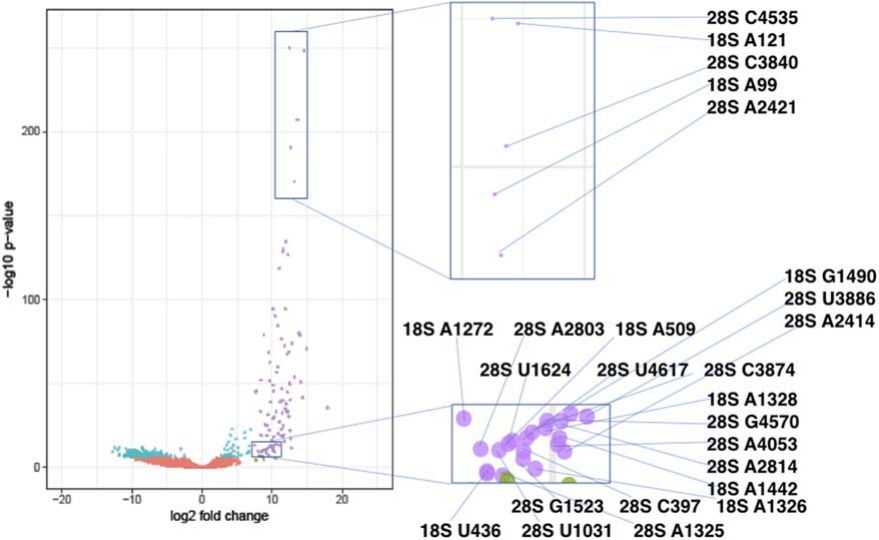
Volcano plot of the −log_10_ *P*-value versus log_2_ fold change in data from human PA1 cells. Each dot represents a single base position in 18S and 28S rRNAs. Base positions were artificially filtered by *P*-values and log_2_ fold changes and color-coded. Red dots represent positions with log_2_ fold change ≤7 and adjusted *P*-value >0.0001. Teal dots represent log_2_ fold change ≤7 and adjusted *P*-value <0.0001. Green dots represent log_2_ fold change >7 and adjusted *P*-value >0.0001. Purple dots represent log_2_ fold change >7 and adjusted *P*-value <0.0001. Positions labeled with purple were determined as highest confidence sites. The zoomed-in views for two regions indicate the actual methylation sites represented by the dots. The Volcano plot was generated using the R package ggplot2 (Heng et al. 2009). RibOxi-seq was used on 7 µg samples of total RNA from PA1 cells. The NextSeq500 150 cycle Mid Output Kit was used in the 75 bp by 75 bp configuration. Across three control samples, the sequencer output was ∼14 million, ∼13 million, and ∼36 million reads for each, while oxidized samples had ∼9 million, ∼10 million, and ∼16 million reads each.

**FIGURE 5.**
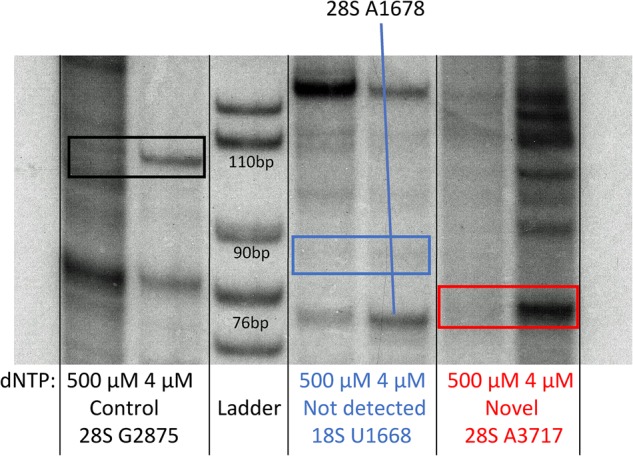
Primer extension analysis. For novel site validation, primers were P^32^ labeled. One microgram of total RNA, 1 µL (10 µM) labeled RT primer, 1 µL 100 µM or 1 µL 10 mM (control) concentrations of dNTPs, 7 µL of water were denatured at 65°C for 5 min, then chilled on ice. An RT Master mix (10 µL per reaction) containing 2 µL 10× RT Buffer, 1 µL (40 U) RNase Out, 1 µL AMV RT (NEB), and 6 µL water was prepared, and added to the RNA/primer mix. Incubation was at 42°C for 45 min. Reactions were ethanol precipitated and resuspended in loading buffer for TBE-PAGE electrophoresis. This experiment was performed for three selected sites from RibOxi-seq: positive control at 28S C1880 (lanes *1*,*2*), known but not detected 18S U1668 (lanes *4*,*5*) and newly detected and not previously reported 28S A3717 (lanes *6*,*7*). The first lane of each set is a negative control where primer extension was performed with a higher dNTP concentration. The second lane of each set was performed at low dNTP concentration to promote polymerase pausing at sites of 2′-OMe.

RibOxi-seq was used to analyze total RNA from the human ovary teratoma-derived PA1 cell line. Site detection was filtered by a combination of log_2_ fold change and adjusted *P*-value from the DESeq2 output. All site-annotations and numbering correspond to the hg19 reference genome. The lists of known sites we used were curated as previously described (Krogh et al. 2016). By applying a cutoff value of log_2_ fold change of >7 and adjusted *P*-value of <0.0001, 39 out of 40 known 18S sites and 60 out of 66 known 28S sites were detected with high confidence (Fig. 4; Supplemental Table S1). The filters were set to correspond to the known sites that have the lowest log_2_ fold changes and the highest *P*-values to allow maximum sensitivity (Supplemental Fig. S2). The number of high confidence sites consisted of 93.3% known sites, which include sites newly found and MS validated by Krogh et al. (2016) using Ribometh-seq. Using such cutoffs, only three novel sites (18S: U354, 28S A1322, and A3717) were found. However, when filters were slightly relaxed to log_2_ fold change of >6 and adjusted *P*-value remained unchanged, eight total potential novel sites were identified (Table 1). Among these candidates, A3717, which displays both a very high log_2_ fold change (∼10) and a low adjusted *P*-value (∼1.5 × 10^−13^), in 28S was validated using a primer extension under restricted dNTP concentration (Fig. 5). Blasting A3717 in combination with surrounding bases within the snoRNA database resulted in two potential snoRNA guide hits, HBII-180B and U37, whose guides are complementary to this region and fulfill criteria for methylation at the fourth or fifth base of the rRNA complementary sequence. Although these two snoRNAs were previously noted to also methylate other positions within 28S, snoRNA-guided methylations of multiple targets is known to occur (van Nues et al. 2011). As only a few novel sites of methylation in rRNAs in human have been characterized within recent years, this result argues that the RibOxi-seq method not only identifies known 2′-OMe sites, but also allows the discovery of new ones. In other experiments using this method we have seen evidence of 2′-OMe in 5.8S rRNA and U snRNAs (data not shown).

**TABLE 1.**
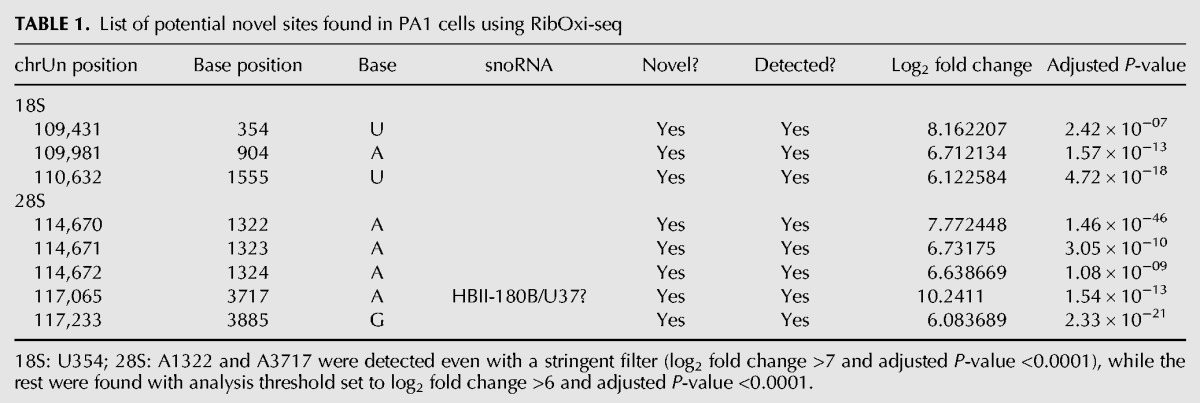
List of potential novel sites found in PA1 cells using RibOxi-seq

### RibOxi-seq results confirm methylation heterogeneity within the same cell line

Among already annotated 2′-*O*-methylation sites, U4497 and G4498 in 28S are positioned immediately adjacent to each other. One limitation of the RibOxi-seq method is that if two sites back to back are both methylated, the site closer to the 5′-end cannot be detected if the other site is fully methylated. This is because Benzonase (as well as other ribonucleases and alkali) cannot cleave at the 3′-site of 2′-OMe, so the 5′-site can never be exposed using our approach. Thus, if G4498 were fully methylated, U4497 would not be detected. Indeed, we did not see U4497, indicating G4498 may be fully methylated. However, another set of back-to-back pairs of 28S sites (U4226 and G4227) were both detected with high confidence. The most probable explanation for our results is that G4227 is only partially methylated, with the unmethylated population allowing the exposure of U4226 (Supplemental Table S1). This result is consistent with the data obtained using Ribometh-seq as well as observations of fractional methylation from primer extension experiments (Maden 1986; Krogh et al. 2016). Such patterns prompted us to consider the possibility that annotated 2′-*O*-methylation sites not detected by our method may be the result of a complete lack of methylation. To test this possibility, radioactive primer extension with low dNTP concentration was used to examine the only missing site in 18S, U1668. As expected, a stop corresponding to that modification site was not detected (Fig. 4). It is interesting to note that this site was also not detected using Ribometh-seq in HeLa cells (Krogh et al. 2016). Further evidence from more cell lines will be required to confirm whether this site is actually modified in other cells or tissues.

### RibOxi-seq requires modest input material but not high sequencing depth

Accurate determination of sites of 2′-OMe using RibOxi-seq relies not only on peak calling of oxidized samples, but also on statistical comparison of signals between oxidized and control lanes. An initial pilot experiment using a small quantity of total RNA in combination with Illumina MiSeq sequencing generated ∼2.5 million reads for each sample (note: the actual number of aligned reads was much lower). Upon examining alignment with 3′-end only reporting, the pattern was strikingly consistent between experiments, with known sites across 18S and 28S rRNAs represented by strong peaks in oxidized samples with corresponding gaps in control samples mapping to the known sites. After single-base differential expression analysis, 36/40 sites in 18S and 54/66 sites in 28S were detected using a filtering strategy similar to that described above. However, there were also more than 30 new sites detected (Supplemental Table S2). Those were likely false positives owing to a lack of enough total available control sample 3′-base counting reads for DESeq2 statistical analysis. Thus, while promising, this pilot experiment was not good enough for accurate peak calling. In our experience, highly sensitive and accurate site detection is achievable at ∼12 million reads (sequencer output) per sample.

Ribose methylation occurs about 1 in every 60 nucleotides (nt) in 28S and 18S rRNA. Under such conditions, one round of β-elimination is sufficient for accurate site detection even at low sequencing depth. However, the occurrence of methylation is very likely to be far lower in RNAs, such as lncRNAs and mRNAs, and no instances of this modification have been reported so far in human, other than in 5′-cap structures. In order to detect mRNA modifications, not only will higher sequencing coverage be required, but also perhaps multiple β-elimination steps to greatly increase the probability of 3′-end 2′-OMe exposure. Also, additional β-elimination can be used on rRNAs if the amount of input total RNA (∼7.5 µg per sample) described in the standard protocol is impossible to obtain. The required starting material can be divided by 2–4 for each round of β-elimination added. For step-by-step instructions on performing additional β-eliminations, please refer to the alternative steps in the Supplemental Data.

### Limitations

We have demonstrated that RibOxi-seq is highly sensitive and accurate. However, several limitations still exist. Although the number of sequencing reads required is significantly lower than that for Ribometh-seq, the input RNA material required is in general somewhat higher, at the micrograms level, with the possibility of reduction to a sub-microgram level if using additional β-elimination steps. Also, as described above, owing to the nature of ribose methylated bases being resistant to nuclease and alkaline hydrolysis, it is difficult to detect adjacent modified bases if the distal base is fully methylated. Further, exposure of methylated bases relies on extensive and random digestion. tRNAs and other RNAs shorter than 100 bp are difficult to study because the sizes of fragments that would need to be generated might be quite small and difficult to examine. Finally, the method at the current stage cannot be used as a quantitative tool to compare methylation intensity between different sites, since linker ligation efficiencies using T4 RNA ligases 1 and 2 have been described to have sequence biases (Raabe et al. 2014). Hence, without first determining ligation efficiencies of linkers to each of A, U, C, and G bases using spike-ins of known 2′-*O*-methylated oligos as internal controls, comparisons between different sites is not yet possible. On the other hand, comparison between different samples at the same site appears to be feasible.

## MATERIALS AND METHODS

### Equipment

Table top centrifuge
Programmable thermal cycler
Heat blocks/water baths
NanoDrop 2000 UV-Vis Spectrophotometer (Thermo Fisher Scientific, ND-2000)
2200 TapeStation (Agilent Technologies, G2964AA)
Qubit 2.0 Fluorometer (Thermo Fisher Scientific, Q32866)
NextSeq 550 System (Illumina)

### Reagents

Seal-Rite 2.0 mL microcentrifuge tube, natural (USA Scientific, 1620-2720)
PA-1 [PA1] cell line (ATCC, CRL-1572)
PureLink RNA Mini Kit (Thermo Fisher Scientific, 12183025)
PureLink DNase Set (Thermo Fisher Scientific, 12185010)
TURBO DNA-free Kit (Thermo Fisher Scientific, AM1907)
Ultra-pure Benzonase (Sigma, E826305KU)
10× Benzonase buffer (store at 4°C)

**Table:** 

10× concentrations	Stock (mM)	Dilution factor	Amount per 10 mL (µL)
500 mM Tris (7.5)	1000	2	5000
100 mM NaCl	5000	50	200
10 mM MgCl_2_	1000	100	100
1 mM EDTA	500	500	20
1 mg/mL BSA	10	10	1000
H_2_0 to 10 mL			3680
3 M sodium acetate pH = 5.2
Ethanol 100%
Ethanol 70%
UltraPure Phenol:Chloroform:Isoamyl Alcohol (25:24:1, v/v) (Thermo Fisher Scientific, 15593031)
Acid-Phenol:Chloroform, pH 4.5 (with IAA, 125:24:1) (Thermo Fisher Scientific, AM9720)
NucAway Spin Columns (Thermo Fisher Scientific, AM10070)
RNA Analysis ScreenTape (Agilent, 5067-5576)
RNA Analysis ScreenTape reagents (Agilent, 5067-5577)
High sensitivity D1000 DNA ScreenTape (Agilent, 5067-5584)
High sensitivity D1000 DNA ScreenTape reagents (Agilent, 5067-5585)
Linear polyacrylamide 10 µg/µL (Mullins Molecular Retrovirology Lab Short protocol)
Sodium meta-periodate (Sigma-Aldrich, 7790-28-5)
Sodium periodate oxidation buffer: 4.375 mM sodium borate, 50 mM boric acid, pH = 8.6
0.2 mL PCR 8-tube FLEX-FREE strip, attached clear flat caps, natural (USA Scientific, 1402-4700)
β-elimination buffer: 33.75 mM sodium borate, 50 mM boric acid, pH = 9.5
T4 Polynucleotide Kinase (NEB, M0201L)
SUPERase• In RNase Inhibitor 20 U/μL (Thermo Fisher Scientific, AM2696)
RNaseOUT Recombinant Ribonuclease Inhibitor (Thermo Fisher Scientific, 10777019)
T4 RNA Ligase 2, truncated KQ (NEB, M0373S)
T4 RNA Ligase 1 (NEB, M0204S)
DMSO 100%
SuperScript III First-Strand Synthesis System (Thermo Fisher Scientific, 18080051)
Sodium hydroxide 1 N
EB buffer: 10 mM Tris-Cl, pH 8.5
Q5 High-Fidelity 2× Master Mix (NEB, M0492S)
Agencourt AMPure XP, 450 mL (Beckman Coulter Life Sciences, A63882)
Qubit dsDNA HS Assay Kit (Thermo Fisher Scientific, Q32854)
NextSeq 500/550 Mid Output v2 Kit (150 cycles) (Illumina, FC-404-2001)
Shrimp alkaline phosphatase (rSAP, NEB, M0371S)

### Oligos

3′ Preadenylated DNA linker (NEB Universal miRNA Cloning Linker, S1315S, dissolve into 50 µM)
  5′-/rApp/CTGTAGGCACCATCAAT/NH2/-3′
5′ RNA linker (50 µM stock)
  5′-/Biosg/rArCrArCrGrArCrGrCrUrCrUrUrCrCrGrArUrCrU-3
Reverse transcription primer (50 µM stock)
  5′-GTGACTGGAGTTCAGACGTGTGCTCTTCCGATCT***RAN***ATTGATGGTGCCTACAG-3′
  (Important: The “*RAN*” represents a customizable randomer sequence that can be used to remove PCR duplicates later in the data analysis. Six-base randomers are used in the experiments, but longer is recommended for higher sequencing depth.)
Illumina compatible barcoded PCR primers
  **i5:** 5′-aatgatacggcgaccaccgagatctacac***BARCODE***acactctttccctacacgacgctcttccgatct-3′
  i7: 5′-caagcagaagacggcatacgagat*BARCODE*gtgactggagttcagacgtgtgctcttccgatct-3′
  (Barcodes are customizable. The protocol is established using paired-end sequencing with dual barcodes. Important: When demultiplexing, the i7 barcode sequence needs to be specified as reverse complement to what is in ***BARCODE***).

It is possible to design longer linker sequences, matching RT primer and PCR primers to increase amplification specificity and efficiency.

All raw and processed data are available at the NCBI Gene Expression Omnibus (GEO) with accession number GSE96999.

### Standard protocol

The library preparation using standard protocol requires around 4–6 d to complete with a moderate work load, factoring out optimization steps. However, the procedure can be stopped whenever an ethanol precipitation is performed and the sample is resuspended into nuclease-free water. Alternatively, samples can also be left precipitating in 100% EtOH under −20°C indefinitely to increase yield. For step-by-step of the protocol, please refer to the details in the detailed protocol in the Supplemental Material.

#### RNA extraction

For cells cultured in 10 cm Petri dishes, a PureLink RNA Mini Kit is used in conjunction with the PureLink on-column DNase set to isolate RNA and remove genomic DNA. Steps for the extraction are detailed in the Purelink Kit's protocol. In case any overexpression system is used, additional post-extraction gDNA removal may be necessary due to an increase in DNA molarity. The TURBO DNA-free Kit has proven effective for such conditions after following its “Rigorous DNase treatment” protocol.

1. Extract total RNA using the PureLink RNA Mini Kit with PureLink on-column DNase.
2. Optional: Use the Turbo DNase Kit to further remove contaminating DNA.

#### RNA fragmentation

Each sample set should have at least one control and one oxidation sample. We recommend a minimum of three technical replicates for each. After completing fragmentation and the subsequent two steps until just before the oxidation procedure, the RNA loss should be ∼50%. It is recommended to use an initial total RNA amount of 45 µg (can be lowered upon further optimization), which will yield about 28 µg of fragmented RNA (results may vary for each laboratory). The amount of fragmented RNA recommended for oxidation is 4–6 µg, and 1 µg for nonoxidation. Three technical replicates for all samples require about 25 µg total. The amount of starting RNA can be significantly lowered if using the alternative procedure during oxidation and β-elimination steps. Such an alternative is also necessary if applying the method on other samples depleted of rRNAs. Refer to the “Alternative steps” section.

3. In a 1.5 mL Eppendorf tube, dilute 45 µg of total RNA into 400 µL with nuclease-free H_2_O.
4. Vortex the mixture and spin for a brief second to collect all liquid at the bottom.
5. Place the tube into a 90°C heat block for 3 min to denature the RNA and immediately place on ice for at least 1 min.
6. Dilute 1 µL stock Benzonase (341 U/µL) into 500 U/mL using 681 µL of 1× Benzonase buffer. (Always dilute fresh prior to using. Do not freeze.)
7. Add 45 µL of 10× Benzonase buffer and 5 µL of diluted Benzonase to the diluted RNA (final RNA concentration: 100 ng/µL). Incubate on ice for 90 min. (This incubation time only serves as a starting reference since it can greatly vary between different laboratories. Perform a time-course experiment to establish optimal incubation time based on desired fragment length.)
8. Perform phenol–chloroform extraction. Add 450 µL acid phenol:chloroform and vortex for 10 sec. Centrifuge at 20,000*g* at RT for 5 min.
9. Transfer about 450 µL of the supernatant into a new set of 2 mL Eppendorf tubes.
10. Ethanol precipitate the RNA. Add 50 µL of 3 M sodium acetate (0.1× volume). Mix well and add 1250 µL of 100% ethanol (2.5× volume).
11. Mix well and place on ice for >30 min to precipitate RNA.
12. Spin at max (>16,000*g*) at 4°C or RT for ∼30 min.
13. Carefully remove the ethanol without dislodging the pellet.
14. Add ≥500 µL 70% ethanol, vortex, and centrifuge for 10 min to wash the pellet.
15. Repeat step 14 once. Carefully remove the ethanol and air dry the pellet for 2 min (excessive drying of the pellet can greatly reduce yield).
16. Resuspend pellet in 100 µL of nuclease-free H_2_O.
17. Use NucAway spin columns following the kit's protocol. (The fragmentation procedure generates many small fragments of <25 nt in length, while fragments of lengths 25 nt and above are desirable. The presence of very small fragments greatly increases RNA molarity and can impact accuracy of concentration measurements. More importantly, these can overwhelm subsequent enzymatic reactions. Thus, it is ideal to filter the RNAs out at this step.)
18. Measure RNA concentration using a Nanodrop.
19. Optional: Examine fragmented RNA size distribution using TapeStation 2200 and RNA Screen Tapes. Ensure the pattern has a strong peak spanning the region from 25 to 150 bp. Once the Benzonase digestion time is optimized for the laboratory, this step can be omitted.
20. Aliquot 1 µg fragmented RNA for each control replicate and store at −80°C until the 3′-end ligation step.
21. Ethanol precipitate 4–6 µg fragmented RNA of each oxidation sample replicate (no wash needed).

Important: See the “Alternative steps” section.

#### Oxidation

To prepare the fragmented RNAs for subsequent elimination of the 3′-end bases to expose ribose methylated bases that are potentially positioned one base upstream, these ends need to be oxidized into dialdehydes using NaIO_4_.

22. Freshly prepare 200 mM NaIO_4_ solution by dissolving 42.78 mg of the NaIO_4_ powder in 1 mL oxidation buffer. Protect the solution from light. Important: This step should be performed while precipitating the fragmented RNA and not earlier.
23. Dissolve RNA pellet in 30 µL oxidation buffer (make sure the pellet is well resuspended) and add 10 µL of prepared NaIO_4_ solution. Mix well and incubate at room temperature protected from light for 45 min (briefly vortex and spin the reaction tube every 10 min to ensure proper resuspension of the pellet).
24. Adjust the volume to 90 µL with nuclease-free H_2_O.
25. Perform ethanol precipitation. (Incubate on ice for >30 min or −20°C overnight. No wash needed.) Important: After adding 100% ethanol for precipitation and centrifugation, white precipitates/film might be seen scattered on the Eppendorf tube wall due to high salt content and a typical pellet might not be visible. This is normal. RNA can be recovered if the orientation of the tube is kept consistent and a pipettor is used to vigorously flush and/or gently scrap the precipitates/film off the wall after adding elimination buffer.

#### β-Elimination

This step catalyzes the leaving of the 3′-end oxidized base to expose potentially 2′-*O*-methylated bases at the 3′-end of the fragments.

26. Add 50 µL of β-elimination buffer to dissolve the pellet. Within 5 min, vigorously vortex, pipette up and down, or invert the tube to further resuspend the oxidized RNA.
27. Spin the tubes briefly and transfer samples into PCR strip tubes. Tubes with individual caps are highly recommended to prevent cross-contamination, especially in later steps where control samples are handled alongside oxidation samples.
28. Using a thermal cycler, incubate at 45°C for 85 min.
29. Recommended: Process samples through NucAway spin columns to remove unwanted small fragments and salts from the samples (β-elimination alkaline conditions which can generate small undesirable fragments).
30. Transfer samples into a new set of 1.5 mL Eppendorf tubes.
31. Ethanol precipitate and resuspend in 22 µL H_2_O as in the previous oxidation step. Addition of 1 µL LPA is highly recommended to help precipitating and visualizing nucleic acids without disrupting subsequent enzymatic reactions.

#### Phosphate removal and oxidation

To oxidize all 3′-ends that are not 2′-*O*-methylated, another NaIO_4_ treatment is needed. However, after β-elimination, RNA fragment 3′-ends will contain a mixture of 3′-phosphates, 2′-phosphates, and/or 2′-3′-phosphates. It is vital to remove these phosphate groups to avoid false positives in the final data representation. T4 PNK is used to remove all three types of phosphates.

32. Transfer to PCR tubes.
33. Remove 3′-, 2′-, and 2′–3′-cyclic phosphate using T4 PNK. (Important: The PNK buffer used must not contain ATP.)

**Table:** 

Components	Volume (µL)
β-Elimination treated RNA	22
2× PNK buffer diluted from the included 10× buffer (pH adjusted to 6.0)	25
SUPERase• In	1
NEB T4 PNK	2
34. Incubate at 37°C for at least 4 h (longer incubation times may be OK and might be beneficial as T4 PNK is an inefficient phosphatase).
35. Phenol–chloroform extract the dephosphorylated RNA and ethanol precipitate (add 1 µL LPA, no wash needed).
36. Freshly prepare 200 mM NaIO_4_ solution by dissolving 42.78 mg of the NaIO_4_ powder in 1 mL oxidation buffer. Protect the solution from light.
37. Dissolve the RNA pellet thoroughly in 30 µL oxidation buffer.
38. Add 10 µL of prepared NaIO_4_ solution. Mix well and incubate at room temperature protected from light for 45 min (briefly vortex and spin reactions every 10 min to further ensure proper resuspension of the pellet).
39. Transfer to a new 1.5 mL Eppendorf tube and ethanol precipitate RNA (incubate on ice for >30 min or −20°C overnight, wash twice with 70% EtOH) and resuspend in 16 µL H_2_O. If white precipitates remain in the solution, do not remove them.
40. Following these steps, the samples are properly oxidized.

#### 3′ DNA linker ligation

Ligation of 3′ linkers to unoxidized RNA 3′-ends will enable selective reverse transcription and thus, enrichment of 2′-*O*-methylated RNA fragments. From this step on, control samples will be subjected to the exact same procedures.

41. Thaw the control samples and dilute each into 8 µL. Transfer into PCR tubes.
42. Transfer 8 µL of each 16 µL oxidized sample into PCR tubes. Store the remaining 8 µL at −80°C as backup.

**Table:** 

Components	Volume (µL)
Oxidized/control RNA	8
3′ DNA linker 50 µM (final 2.5 µM)	1
RNaseOUT	1
50% PEG8000	7
10× t4 RNA ligase buffer	2
T4 RNA ligase 2 truncated kq	1
43. Ligation of 3′ linker (prepare the reagents in a way that can be properly mixed, as the amount of PEG 8000 added can make it difficult).
44. Incubate the reaction in thermal cycler at 16°C overnight for 18 h.

#### Anneal RT primer

Any 3′ linker not ligated in the previous step will still be freely available for ligation in the samples. To ensure the 5′ RNA linker ligation attaches the RNA linkers to sample RNA fragments but not to the free 3′ linkers during the next step, the RT primer is annealed first.

45. Add 1 µL of the 50 µM RT primer and 69 µL nuclease-free water to each sample.
46. Incubate in thermal cycler with the following program:
  - 90°C for 2 min
  - 65°C for 10 min
  - 4°C for 1 min
47. Phenol–chloroform extract and ethanol precipitate (add 1 µL of LPA) each sample and resuspend in 11 µL H_2_O.

#### 5′ RNA linker ligation

The double-stranded structures resulting from annealing the RT primers and free 3′ linkers will prevent them from being ligated to the 5′ RNA linkers (Munafó and Robb 2010).

48. Thaw 50 µM RNA linker from −80°C and transfer (number of samples) *1.3 µL into a PCR tube.
49. Denature RNA linker at 72°C for 2 min and return to ice.
50. Prepare the following ligation reaction.

**Table:** 

Components	Volume (µL)
Annealed RNA	11
5′RNA linker 50 µM (final 2.5 µM)	1
100% DMSO	2
10× t4 RNA ligase buffer	2
10 mM ATP	2
RNaseOUT	1
T4 RNA ligase 1	1
51. Incubate at 25°C for 1 h, then terminate reaction at 65°C for 15 min.

#### Reverse transcription

Because the RT primer has been annealed in the previous step, the RT reaction can proceed without addition of primer. During this step, cDNA is synthesized. At the same time, random-hexamer sequences built into the RT primer are also incorporated into the cDNA library. These will allow the removal of PCR duplicates later during data treatment.

52. Prepare RT reactions to generate a cDNA library using the following setup with SuperScript III included reagents.

**Table:** 

Components	Volume (µL)
Ligated RNA	19
SuperScript III RT buffer	4
MgCl_2_	8
DTT	4
dNTP mix	2
RNaseOUT	1
SS III enzyme	2
53. Incubate the reactions in thermal cycler following the kit's protocol.
54. Hydrolyze remaining RNAs by adding 4.4 µL of 1 N NaOH and incubate at 98°C for 20 min.
55. Add 22 µL 200 mM Tris-HCl PH = 7.0 to neutralize the PH.
56. Use Ampure XP beads at 0.8:1 ratio (add 53 µL Ampure XP solution). Incubate for 5 min to let beads bind cDNA of sizes 250 bp and above.
57. Transfer supernatant to new tubes (discard beads) and add an additional 67 µL Ampure XP solution to make the ratio 1:1.8. Finish Ampure XP purification.
58. Elute using EB buffer.

#### PCR amplification of cDNA library

Illumina i5 and i7 PCR primer sequences have sequences complementary to the 5′ RNA linker and RT primer sequences. This allows direct PCR amplification of the cDNA library. Periodate oxidation enrichment in previous steps results in a cDNA library of very low complexity. Hence it necessitates additional amplification cycles compared to construction of other types of sequencing libraries (∼35 cycles versus ∼12 cycles). The strandedness of the final library is second-strand similar to the library prepared using the Illumina ligation method.

**Table:** 

Components	Volume (µL)
cDNA	8.5
I5 primer	2
I7 primer	2
Q5 2× master mix	12.5

59. Prepare NEB Q5 PCR reactions.
60. Incubate in the thermal cycler using the following program modified from the Q5 protocol.

**Table:** 

Step	Temp	Time
Initial denaturation	98°C	30 sec
One cycle	98°C	5–10 sec
	55°C	10–30 sec
	72°C	20–30 sec/kb
32 cycles	98°C	5–10 sec
	62°C	10–30 sec
	72°C	20–30 sec/kb
Final extension	72°C	2 min
Hold	4–10°C
61. Add 25 µL of AmpureXP to achieve a 1:1 ratio to select for fragment of sizes ∼200 bp and above, reducing the amount of non-insert fragments (Illumina i5 sequence: ∼70 bp, Illumina i7 sequence: ∼66, total non-insert product: ∼136 bp).
62. Purify libraries following the AmpureXP protocol. Resuspend each sample in 15 µL with Illumina RSB (resuspension buffer).

### Library quantification

63. Use TapeStation and DNA Screen Tape to visualize library size distributions. It should have a distribution around 200 bp. Even though stringent steps have been taken to avoid non-insert PCR products, they may still be present and are expected.
64. Accurately measure library concentrations using the Qbit Fluorometer and dsDNA High Sensitivity Reagent Kit (follow Qbit protocol).
65. Calculate library molarity using sizes and concentrations measured.

#### Sequencing

The NextSeq 500 (or MiSeq) 150 cycle Mid Output Kit is used in this experiment with 75 bp by 75 bp configuration.

66. Prepare and load the libraries for the NextSeq 500 sequencer following the established protocol. Important: Make sure PhiX phage DNA comprises at least 30% of the total library if the Riboxi-seq samples are the only samples being sequenced because of the nature of extremely low diversity amplicon sequencing. The final loading concentration used in this experiment is 1.5 pM, which is slightly lower than the 1.8 pM from the protocol.

### Data treatment

67. Remove read-through sequences. Because of the nature of the sequencing library preparation, many fragments will have insert sizes significantly smaller than the 75 bp length sequenced by the sequencer. The resulting read-through sequences will greatly impact alignment reliability. Cutadapt (Martin 2011), a Python package, is used to first remove read-through sequences at the 3′-end for both read 1 and read 2. An in-house script has been used to compare randomer sequences and ∼5 bp RNA sequences to determine and collapse PCR duplicates on read 2. Finally, we used cutadapt to remove the randomers and linker sequence from 5′-ends of read 2. The resulting “.fastq” files can then be used for alignment.
68. TopHat2 is used to align the reads. The following options are supplied in addition to the basic command: -p 12 -r 10 -N 1 - -b2-L 10 - -segment-length 15 - -segment-mismatches 1 - - library-type fr-secondstrand - -no-mixed - -no-discordant -T - -no-coverage-search -G rRNA_annotation.gtf - - no-novel-juncs. The annotation file used consists of chrUn coordinates, which contain two sets of 18S and 28S rRNAs, extracted from the hg19 index (Kim et al. 2013).
69. The “accepted_hits.bam” for each sample are sorted using SAMtools and converted to “.BED” files using BEDtools (Heng et al. 2009; Quinlan and Hall 2010).
70. The third column of the “.BED” file represents the starting and ending positions (with respect to 5′- and 3′-ends) of each read, while the sixth column indicates sense or anti-sense. We counted the number of reads with 3′-end alignment for each position corresponding to 18S and 28S rRNA and generated a count table for each sample (only sense reads are taken, since the two rRNAs we aligned to the reference sequence were transcribed from the sense strand).
71. The count data files were then imported into DESeq2 for differential analysis per base position (Love et al. 2014).

### Alternative steps

This procedure should be performed between steps 21–22 and between steps 34–35 in the original standard protocol for low starting RNA material or for samples where rRNA has been depleted.

#### Oxidation

1. Dissolve the RNA pellet in 30 µL oxidation buffer (make sure the pellet is well resuspended) and add 10 µL of prepared NaIO_4_ solution. Mix well and incubate at room temperature protected from light for 45 min (briefly vortex and spin the reaction tube every 10 min to ensure proper resuspension of the pellet).
2. Add 10 µL sodium acetate and adjust the volume to 100 µL with nuclease-free H_2_O.
3. Perform ethanol precipitation (no wash needed).

#### β-Elimination

4. Add 50 µL of β-elimination buffer to dissolve the pellet. Within 5 min, try as much as possible to vortex, pipetting up and down, or inverting the tube to further resuspend the oxidized RNA.
5. Spin the tubes briefly and transfer samples into PCR strip tubes. (Tubes with individual caps are highly recommended to prevent cross-contamination, especially in later steps where control samples are handled alongside with oxidation samples.)
6. Using a thermal cycler, incubate at 45°C for 85 min.
7. Transfer samples into a new set of 1.5 mL Eppendorf tubes.
8. Ethanol precipitate and resuspend in 31 µL H_2_O as in the previous oxidation step. Dissolve thoroughly.
9. Transfer to PCR tubes.

#### Dephosphorylate 3′-ends

10. Prepare the following reaction:

**Table:** 

Components	Volume (µL)
RNA	31
CutSmart Buffer (10×)	4
rSAP (NEB)	4
RNaseOUT	1
11. Incubate at 37°C for 30 min. Stop reaction by heat-inactivation at 65°C for 5 min.
12. Phenol–chloroform extraction and ethanol precipitate (add 1 µL LPA) RNA into pellet.
13. Repeat alternative steps 1–12 as needed (may require optimization).
14. Continue to step 22 in the standard protocol.
15. The following steps to phosphorylate fragment 5′-end are required between steps 34 and 35 of the standard protocol because rSAP removes both 5′ and 3′ phosphates.
16. Ethanol precipitate reaction and resuspend with 29 µL H_2_O.

**Table:** 

Components	Volume (µL)
RNA	29
10× NEB PNK Buffer	4
10 mM ATP	4
T4 PNK	2
RNaseOUT	1
17. Prepare the following reaction:
18. Incubate at 37°C for 60 min.
19. Continue to step 35 in the standard protocol.

## SUPPLEMENTAL MATERIAL

Supplemental material is available for this article.

## Supplementary Material

Supplemental Material
