# Supplementary material for: High-throughput and site-specific identification of 2′-O-methylation sites using ribose oxidation sequencing (RibOxi-seq)
Source: RNA. 2017 Aug;23(8):1303–14. doi: 10.1261/rna.061549.117 (PMC5513074; doi:10.1261/rna.061549.117)
Supplement: Supplemental Material [file supp_23_8_1303__index.html]

High-throughput and site-specific identification of 2′-O-methylation sites using ribose oxidation sequencing (RibOxi-seq) — Supplemental Material 

# High-throughput and site-specific identification of 2′-*O*-methylation sites using ribose oxidation sequencing (RibOxi-seq)

## Supplemental Material

- Supplemental\_Material.docx
