# Supplementary material for: High-throughput and site-specific identification of 2′-O-methylation sites using ribose oxidation sequencing (RibOxi-seq)
Source: RNA. 2017 Aug;23(8):1303–14. doi: 10.1261/rna.061549.117 (PMC5513074; doi:10.1261/rna.061549.117)
Supplement: Supplemental Material [file supp_061549.117_Supplemental_Material.docx]

**Table S1**

Full list of 2’-O methylation sites within 18S and 28S rRNAs in PA1 cells determined by filtering DESeq2 analysis output (libraries sequenced on Illumina nextseq) using log2FC (>6) and adjusted p value (0.0001). Positions highlighted in blue are sites that are annotated but not detected using our method.

18S:

| chrUn coordinate | Base position | Base | snoRNAs | Novel? | Detected? | log2FC | adjusted p value |
| --- | --- | --- | --- | --- | --- | --- | --- |
| 109104 | 27 | A | U27 | no | yes | 17.92943 | 3.43E-36 |
| 109176 | 99 | A | U57 | no | yes | 12.66704 | 1.68E-191 |
| 109193 | 116 | U | U42A/B | no | yes | 12.43167 | 2.18E-69 |
| 109198 | 121 | U | mgh18S-121/Z17B | no | yes | 14.57216 | 3.9E-249 |
| 109236 | 159 | A | U45A/C | no | yes | 9.515039 | 6.76E-08 |
| 109243 | 166 | A | U44 | no | yes | 12.78353 | 3.81E-12 |
| 109249 | 172 | U | U45A/B | no | yes | 10.19354 | 1.51E-11 |
| 109251 | 174 | C | SNORD45C | no | yes | 11.79013 | 5.83E-73 |
| 109505 | 428 | U | HBII-202 | no | yes | 9.514961 | 5.07E-69 |
| 109513 | 436 | G | HBII-429 | no | yes | 7.66968 | 1.64E-45 |
| 109539 | 462 | C | U14A/B | no | yes | 12.0917 | 9.85E-34 |
| 109545 | 468 | A | SNORD83A/68 | no | yes | 12.55015 | 4.02E-50 |
| 109561 | 484 | A | U16 | no | yes | 11.59956 | 1.64E-25 |
| 109586 | 509 | G | HBII-95/B | no | yes | 7.985352 | 3.07E-10 |
| 109589 | 512 | A | HBII-234 | no | yes | 12.83362 | 1.98E-74 |
| 109594 | 517 | C | U56 | no | yes | 14.91788 | 2.85E-71 |
| 109653 | 576 | A | HBII-336 | no | yes | 13.86214 | 9.17E-81 |
| 109667 | 590 | A | U62A/B | no | yes | 10.33406 | 6.34E-10 |
| 109679 | 601 | G | HBII-251/U103/B | no | yes | 12.56987 | 1.75E-22 |
| 109704 | 627 | U | HBII-135 | no | yes | 9.429529 | 3.18E-12 |
| 109721 | 644 | G | U54 | no | yes | 12.67708 | 9.15E-18 |
| 109745 | 668 | A | U36A/B | no | yes | 11.75379 | 3.81E-16 |
| 109760 | 683 | G | HBII-108/B | no | yes | 14.08851 | 2.06E-51 |
| 109874 | 797 | C | ZL107/GGgCD20 | no | yes | 9.685468 | 2.90E-63 |
| 109876 | 799 | U | U105/B | no | yes | 10.46362 | 5.47E-06 |
| 109944 | 867 | G | HBII-419 | no | yes | 10.21481 | 2.95E-46 |
| 110108 | 1031 | A | U59A/B | no | yes | 7.713374 | 2.73E-05 |
| 110349 | 1272 | C | HBII-142 | no | yes | 7.509314 | 7.58E-14 |
| 110365 | 1288 | U | HBII-55 | no | yes | 12.14759 | 4.76E-68 |
| 110403 | 1326 | U | U33 | no | yes | 8.592145 | 4.66E-07 |
| 110405 | 1328 | G | U32A | no | yes | 8.730437 | 1.33E-06 |
| 110460 | 1383 | A | SNORD30 | no | yes | 11.30044 | 1.58E-19 |
| 110468 | 1391 | C | U28 | no | yes | 14.38748 | 2.14E-42 |
| 110519 | 1442 | U | U61 | no | yes | 8.874819 | 3.31E-11 |
| 110524 | 1447 | G | SNORD127 | no | yes | 11.02978 | 3.65E-119 |
| 110567 | 1490 | G | U25 | no | yes | 9.173948 | 4.75E-09 |
| 110745 | 1668 | U | Unknown | no | no |  |  |
| 110755 | 1678 | A | U82 | no | yes | 12.47255 | 5.01E-51 |
| 110780 | 1703 | C | U43 | no | yes | 11.16467 | 9.52E-40 |
| 110881 | 1804 | U | U20 | no | yes | 13.96577 | 2.07E-79 |

28S:

| chrUn coordinate | Base position | Base | snoRNAs | Novel? | Detected? | log2FC | adjusted p value |
| --- | --- | --- | --- | --- | --- | --- | --- |
| 113745 | 397 | A | U26 | no | yes | 8.330468 | 5.02E-34 |
| 113747 | 399 | A | U81 | no | yes | 12.62798 | 5.39E-41 |
| 114663 | 1315 | G | U21 | no | yes | 9.655468 | 2.27E-52 |
| 114670 | 1322 | A |  | yes | yes | 7.772448014 | 1.46E-46 |
| 114673 | 1325 | A | U18A/B/C | no | yes | 11.56306 | 1.42E-40 |
| 114687 | 1339 | C | U104 | no | yes | 8.451424 | 8.58E-20 |
| 114869 | 1521 | G | snR39B | no | no |  |  |
| 114871 | 1523 | A | U32A/B/U51 | no | yes | 10.86955 | 1.06E-20 |
| 114881 | 1533 | A | U77/U80 | no | yes | 8.162373 | 1.46E-07 |
| 114972 | 1624 | G | U80 | no | yes | 11.99951 | 1.80E-38 |
| 115107 | 1759 | G |  | no | yes | 9.31923 | 1.37E-25 |
| 115218 | 1870 | A | U38A/B | no | yes | 10.96471 | 4.15E-14 |
| 115228 | 1880 | C |  | no | yes | 10.55289 | 7.79E-34 |
| 115639 | 2291 | C | U48 | no | no |  |  |
| 115698 | 2350 | C | U24 | no | yes | 9.587046 | 8.89E-23 |
| 115710 | 2362 | A | U76 | no | no |  |  |
| 115712 | 2364 | C | U24 | no | yes | 11.3781 | 2.84E-77 |
| 115748 | 2400 | A | HBII-202 | no | yes | 9.007025 | 2.18E-26 |
| 115762 | 2414 | U | ZL5/6/SNORD143/144 | no | yes | 11.36035 | 2.97E-23 |
| 115769 | 2421 | C | mgh28S-2409 | no | yes | 13.18647 | 6.38E-171 |
| 115771 | 2423 | G | mgh28S-2411 | no | yes | 10.01012 | 3.45E-17 |
| 116134 | 2786 | A | HBII-420 | no | yes | 7.649203 | 6.31E-05 |
| 116151 | 2803 | C | U55 | no | yes | 8.838489 | 1.27E-79 |
| 116162 | 2814 | A | U95 | no | yes | 12.29032 | 7.88E-34 |
| 116171 | 2823 | C | U95 | no | yes | 10.82754 | 1.51E-60 |
| 116184 | 2836 | U | U34 | no | yes | 11.68902 | 7.29E-20 |
| 116208 | 2860 | C | U50 | no | yes | 11.05747 | 9.81E-28 |
| 116223 | 2875 | G | U50 | no | yes | 9.26956 | 1.08E-19 |
| 116974 | 3626 | G |  | no | yes | 11.41354 | 1.91E-47 |
| 117049 | 3700 | C | HBII-180A/B/C | no | yes | 11.8941 | 1.28E-35 |
| 117065 | 3717 | A | HBII-180B | yes | yes | 10.24109708 | 1.54E-13 |
| 117067 | 3719 | A | U37 | no | no |  |  |
| 117071 | 3723 | A | U36C | no | yes | 10.50822 | 2.07E-14 |
| 117091 | 3743 | G | HBII-276 | no | yes | 6.466373 | 5.50E-09 |
| 117107 | 3759 | A | U46 | no | yes | 8.50116 | 4.32E-10 |
| 117132 | 3784 | A | U15A/B | no | yes | 10.15055 | 8.93E-11 |
| 117139 | 3791 | G | SNORD15A | no | yes | 10.0732 | 1.37E-29 |
| 117155 | 3807 | C | mgU6-77 | no | yes | 6.963681 | 3.84E-23 |
| 117165 | 3817 | U | ACA48/HBI-43 | no | yes | 12.21898 | 1.79E-45 |
| 117172 | 3824 | A | U30 | no | yes | 11.44786 | 5.37E-33 |
| 117177 | 3829 | A | U79 | no | yes | 13.18303 | 1.53E-54 |
| 117188 | 3840 | C | U74 | no | yes | 13.59508 | 9.18E-208 |
| 117214 | 3866 | A | HBII-316 | no | yes | 10.11454 | 4.60E-95 |
| 117216 | 3868 | C | U53 | no | yes | 11.55245 | 3.30E-129 |
| 117234 | 3886 | C | U47 | no | yes | 9.185954 | 5.57E-10 |
| 117246 | 3898 | G | HBII-99/B | no | yes | 10.27247 | 1.36E-30 |
| 117272 | 3924 | U | U52 | no | yes | 9.274559 | 1.51E-11 |
| 117291 | 3943 | G | HBII-82/B | no | yes | 12.25001 | 1.73E-127 |
| 117391 | 4043 | G | U102 | no | no |  |  |
| 117401 | 4053 | C | U75 | no | yes | 8.835343 | 1.99E-05 |
| 117543 | 4195 | G | U31 | no | yes | 12.39906 | 5.00E-31 |
| 117574 | 4226 | U | U58C | no | yes | 9.428109 | 4.73E-32 |
| 117575 | 4227 | G | U58A/B/C | no | yes | 10.08335 | 1.34E-49 |
| 117653 | 4305 | U | U41 | no | yes | 11.59332 | 8.11E-19 |
| 117717 | 4369 | G | U60 | no | yes | 9.848911 | 1.48E-13 |
| 117739 | 4391 | G | snR38A/B/C | no | yes | 10.2434 | 6.89E-19 |
| 117803 | 4455 | C | U49A/B | no | yes | 11.95609 | 2.11E-135 |
| 117841 | 4493 | G | HBII-210 | no | yes | 10.71001 | 3.04E-57 |
| 117845 | 4497 | U | SNORD62A/B | no | no |  |  |
| 117846 | 4498 | G | SNORD62A/B | no | yes | 10.83552 | 5.26E-85 |
| 117870 | 4522 | A | U29 | no | yes | 9.798517 | 1.09E-12 |
| 117883 | 4535 | C | U35A/B | no | yes | 12.49586 | 9.45E-251 |
| 117918 | 4570 | A | U63 | no | yes | 8.764135 | 7.07E-11 |
| 117937 | 4589 | A | SNORD119 | no | yes | 11.60263 | 8.32E-131 |
| 117965 | 4617 | G | HBII-296A/B | no | yes | 8.407214 | 1.45E-52 |
| 117967 | 4619 | U | HBII-240 | no | yes | 10.46333 | 1.10E-90 |
| 117970 | 4622 | G | U78 | no | yes | 11.90306 | 4.03E-95 |
| 117984 | 4636 | G | SNORD121A/B | no | yes | 9.767133 | 1.42E-39 |

**Table S2**

Methylation site analysis using the same pipeline in a pilot experiment (low starting material, low sequencing reads) sequenced on Illumina MiSeq. After setting cutoffs of log2FC >7 and adjusted p value <0.0001 similar to what we did with the nextseq experiment, we still obtained more than 30 ‘novel’ sites with high significance values. However, this may likely be due to a lack of more thorough coverage in the control sample for accurate statistical calculations.

| Base position | log2FC | Adjusted p value | Gene |
| --- | --- | --- | --- |
| 27 | 10.55443 | 2.43E-07 | 18S |
| 99 | 8.799656 | 2.35E-73 | 18S |
| 116 | 10.71197 | 1.22E-72 | 18S |
| 121 | 13.71922 | 1.89E-18 | 18S |
| 159 | 9.326041 | 3.66E-67 | 18S |
| 166 | 9.931337 | 1.67E-49 | 18S |
| 172 | 11.95536 | 1.18E-21 | 18S |
| 174 | 8.607579 | 1.46E-86 | 18S |
| 462 | 10.53898 | 1.75E-170 | 18S |
| 468 | 9.619072 | 6.40E-135 | 18S |
| 484 | 12.23966 | 3.81E-157 | 18S |
| 509 | 11.07514 | 1.34E-17 | 18S |
| 512 | 11.0282 | 1.35E-33 | 18S |
| 517 | 9.94368 | 6.74E-116 | 18S |
| 576 | 12.1825 | 9.00E-57 | 18S |
| 590 | 10.51372 | 1.80E-117 | 18S |
| 591 | 9.689266 | 2.59E-22 | 18S |
| 627 | 14.76365 | 2.10E-22 | 18S |
| 644 | 12.03113 | 6.26E-30 | 18S |
| 668 | 11.57821 | 4.11E-23 | 18S |
| 683 | 13.17586 | 9.80E-18 | 18S |
| 799 | 8.277938 | 5.45E-52 | 18S |
| 867 | 10.73573 | 1.20E-81 | 18S |
| 938 | 8.206069 | 2.58E-14 | 18S |
| 1030 | 9.074891 | 1.26E-12 | 18S |
| 1031 | 15.18313 | 2.59E-23 | 18S |
| 1032 | 9.132846 | 2.41E-19 | 18S |
| 1082 | 8.33019 | 2.34E-10 | 18S |
| 1085 | 8.214006 | 1.65E-11 | 18S |
| 1288 | 9.835085 | 5.79E-103 | 18S |
| 1326 | 13.35978 | 5.35E-45 | 18S |
| 1328 | 10.34774 | 1.92E-63 | 18S |
| 1383 | 10.46936 | 3.02E-89 | 18S |
| 1391 | 12.86007 | 6.87E-60 | 18S |
| 1442 | 11.33767 | 4.08E-192 | 18S |
| 1447 | 9.466752 | 8.73E-49 | 18S |
| 1483 | 10.49649 | 4.74E-40 | 18S |
| 1490 | 13.62181 | 6.15E-83 | 18S |
| 1623 | 10.16188 | 8.78E-25 | 18S |
| 1678 | 13.80539 | 3.05E-116 | 18S |
| 1703 | 11.47862 | 1.85E-23 | 18S |
| 1804 | 10.08296 | 6.32E-166 | 18S |
| 41 | 11.37737 | 4.99E-54 | 28S |
| 43 | 8.531992 | 5.95E-80 | 28S |
| 46 | 9.737946 | 3.89E-05 | 28S |
| 54 | 8.930426 | 1.44E-60 | 28S |
| 343 | 11.36113 | 6.20E-11 | 28S |
| 365 | 11.52151 | 9.24E-14 | 28S |
| 366 | 10.65676 | 1.84E-11 | 28S |
| 368 | 9.062115 | 1.33E-13 | 28S |
| 397 | 12.99325 | 5.60E-209 | 28S |
| 399 | 13.04195 | 1.21E-133 | 28S |
| 1325 | 13.65446 | 2.37E-18 | 28S |
| 1339 | 10.17321 | 6.07E-40 | 28S |
| 1521 | 10.446 | 2.96E-34 | 28S |
| 1523 | 10.74807 | 4.65E-78 | 28S |
| 1533 | 8.536363 | 1.18E-95 | 28S |
| 1624 | 13.90523 | 7.16E-77 | 28S |
| 1653 | 8.293689 | 7.55E-07 | 28S |
| 1722 | 8.12126 | 1.95E-28 | 28S |
| 1759 | 13.34798 | 2.22E-43 | 28S |
| 1870 | 15.20598 | 2.86E-58 | 28S |
| 1880 | 9.022158 | 5.03E-77 | 28S |
| 2032 | 8.529392 | 2.72E-13 | 28S |
| 2340 | 10.40612 | 2.16E-15 | 28S |
| 2346 | 8.85539 | 2.11E-19 | 28S |
| 2350 | 11.01845 | 1.95E-242 | 28S |
| 2363 | 9.173699 | 7.04E-21 | 28S |
| 2364 | 10.97399 | 2.53E-121 | 28S |
| 2365 | 8.528839 | 2.22E-09 | 28S |
| 2414 | 8.889401 | 1.54E-165 | 28S |
| 2421 | 11.40437 | 3.87E-188 | 28S |
| 2423 | 11.7927 | 9.03E-230 | 28S |
| 2786 | 11.87395 | 6.44E-144 | 28S |
| 2788 | 10.68347 | 7.55E-12 | 28S |
| 2797 | 11.00513 | 1.10E-10 | 28S |
| 2803 | 10.81631 | 8.74E-74 | 28S |
| 2814 | 10.73284 | 2.62E-49 | 28S |
| 2823 | 13.54179 | 2.76E-46 | 28S |
| 2836 | 12.53423 | 3.07E-60 | 28S |
| 2856 | 8.745453 | 1.97E-08 | 28S |
| 2860 | 11.14712 | 4.92E-164 | 28S |
| 2875 | 10.92658 | 4.75E-124 | 28S |
| 3214 | 8.058361 | 4.97E-14 | 28S |
| 3626 | 9.908804 | 6.27E-105 | 28S |
| 3700 | 10.79715 | 1.29E-111 | 28S |
| 3717 | 9.718723 | 1.41E-64 | 28S |
| 3723 | 10.61679 | 1.37E-30 | 28S |
| 3743 | 8.702421 | 5.39E-199 | 28S |
| 3759 | 10.21367 | 1.58E-172 | 28S |
| 3774 | 8.03047 | 7.37E-38 | 28S |
| 3784 | 15.41124 | 2.89E-29 | 28S |
| 3785 | 8.189249 | 5.13E-49 | 28S |
| 3791 | 15.82264 | 1.56E-106 | 28S |
| 3798 | 13.85013 | 6.70E-20 | 28S |
| 3800 | 10.37691 | 3.41E-49 | 28S |
| 3801 | 9.734033 | 9.62E-37 | 28S |
| 3802 | 14.46588 | 5.12E-20 | 28S |
| 3803 | 13.22142 | 1.53E-13 | 28S |
| 3804 | 13.62985 | 3.84E-19 | 28S |
| 3805 | 12.40083 | 8.30E-26 | 28S |
| 3806 | 15.98669 | 1.49E-26 | 28S |
| 3807 | 15.42724 | 1.06E-24 | 28S |
| 3808 | 14.0221 | 1.70E-19 | 28S |
| 3810 | 9.435787 | 9.83E-11 | 28S |
| 3812 | 12.78899 | 1.30E-14 | 28S |
| 3817 | 9.351792 | 1.69E-14 | 28S |
| 3824 | 8.077241 | 7.37E-30 | 28S |
| 3829 | 8.694021 | 5.42E-70 | 28S |
| 3840 | 11.11597 | 9.49E-141 | 28S |
| 3866 | 10.58732 | 7.14E-37 | 28S |
| 3868 | 11.93004 | 2.03E-57 | 28S |
| 3886 | 11.47013 | 2.56E-86 | 28S |
| 3898 | 12.94397 | 2.14E-14 | 28S |
| 3924 | 10.33772 | 2.88E-207 | 28S |
| 3935 | 11.1246 | 6.70E-20 | 28S |
| 3936 | 10.21104 | 9.92E-19 | 28S |
| 3943 | 9.934799 | 3.88E-110 | 28S |
| 4053 | 13.07942 | 7.75E-32 | 28S |
| 4195 | 11.21392 | 2.23E-15 | 28S |
| 4227 | 12.64137 | 1.43E-28 | 28S |
| 4262 | 9.000383 | 1.25E-28 | 28S |
| 4270 | 9.746785 | 7.96E-14 | 28S |
| 4305 | 12.75388 | 4.20E-292 | 28S |
| 4369 | 10.23483 | 5.17E-43 | 28S |
| 4391 | 14.12186 | 2.94E-112 | 28S |
| 4395 | 8.132709 | 6.18E-31 | 28S |
| 4455 | 12.07974 | 2.15E-115 | 28S |
| 4464 | 8.808197 | 5.19E-34 | 28S |
| 4493 | 10.9663 | 2.70E-07 | 28S |
| 4498 | 9.873865 | 1.74E-126 | 28S |
| 4516 | 13.21483 | 4.06E-18 | 28S |
| 4518 | 11.7669 | 4.35E-14 | 28S |
| 4521 | 13.89199 | 2.02E-18 | 28S |
| 4522 | 10.07951 | 4.29E-07 | 28S |
| 4535 | 8.31011 | 4.69E-24 | 28S |
| 4622 | 11.03496 | 2.30E-07 | 28S |
| 4636 | 10.44255 | 8.65E-41 | 28S |
| 4938 | 10.97668 | 1.68E-10 | 28S |

**Figure S1**

Visualization of digestion of a test oligo for confirmation whether Benzonase cleaves at 2’-O methylated bases. The 50-mer oligo was designed to have random phosphorythiolated DNA bases across its entire length except for the central 4 bases. The 4 bases highlighted in red are 2’-O methylated RNA bases.

Gel electrophoresis of digestion under the same conditions as described in the detailed protocol is shown below: If Benzonase were able to cleave at any of the 2’-O methylated bases, a band at ~25bp should appear. However, there is no detectable difference between reactions with or without Benzonase, indicating that Benzonase does not cut at 2’-O methylated bases.


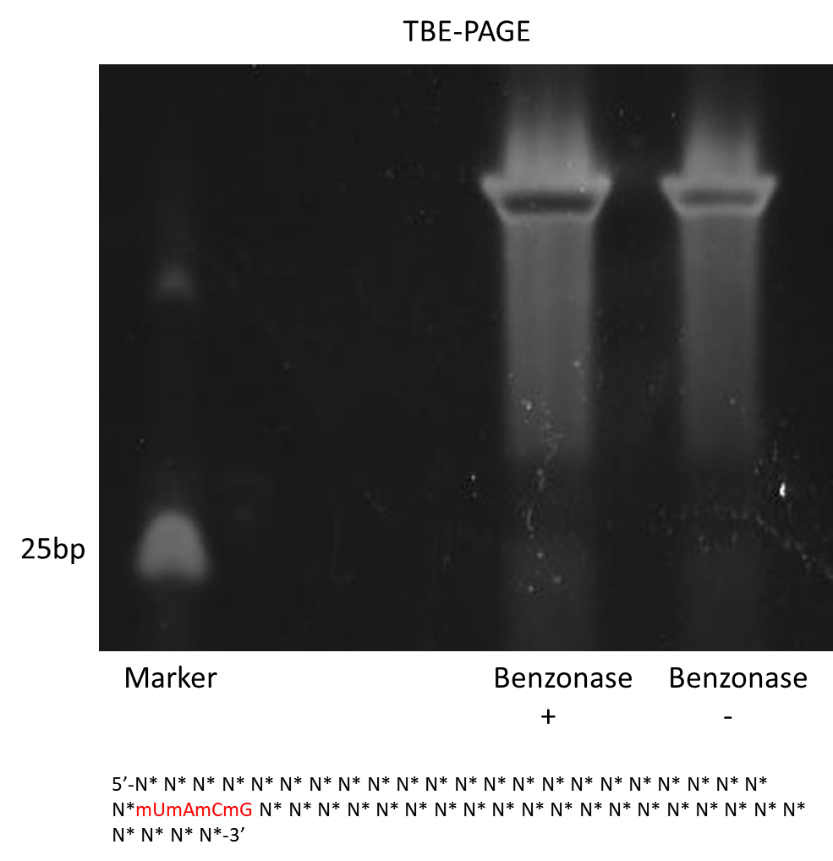


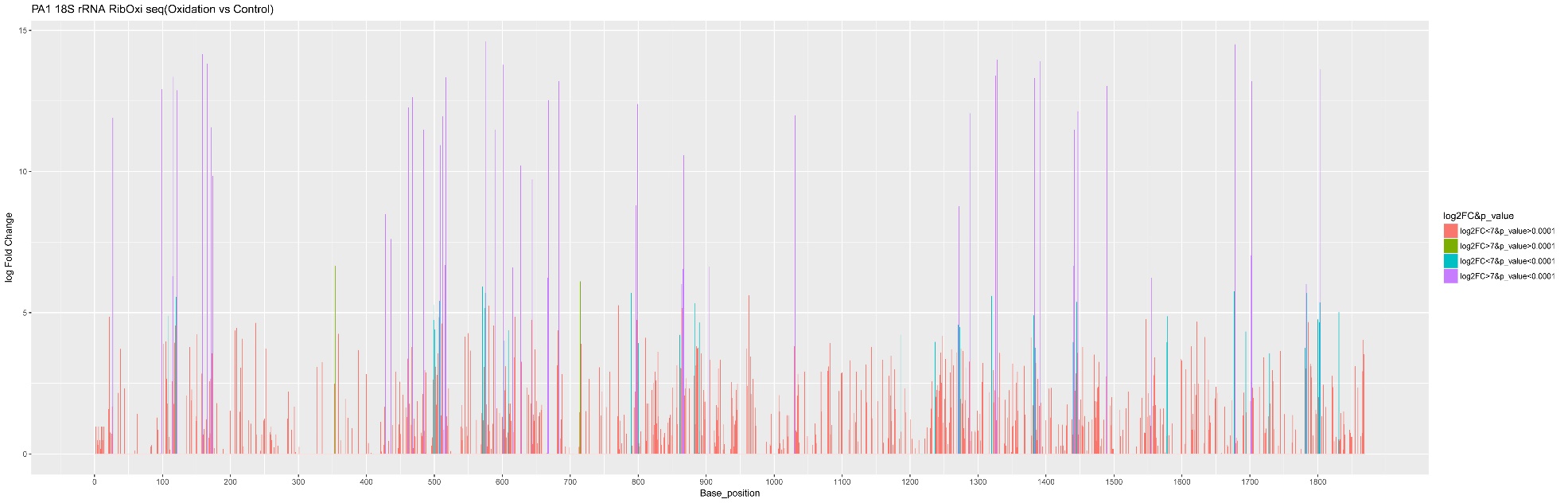


**Figure S2**

Plotting used the same data as shown in in Figure 4 but in bar plot style to indicate filtering choice. Color code is the same as described in Figure 4.

**Primer sequences for radioactive dNTP concentration dependent primer extension:**

LSU C1880 (positive control):

5’-ATGGCCACCGTCCTGCT-3’

SSU U1668 (not detected):

5’-ATCCGAGGGCCTCACTA-3’

LSU A3717 (Novel):

5’-GGCATTTGGCTACCTTA-3’
